# Cox17 Protein Is an Auxiliary Factor Involved in the Control of the Mitochondrial Contact Site and Cristae Organizing System*

**DOI:** 10.1074/jbc.M115.645069

**Published:** 2015-04-27

**Authors:** Magdalena Chojnacka, Agnieszka Gornicka, Silke Oeljeklaus, Bettina Warscheid, Agnieszka Chacinska

**Affiliations:** From the ‡ International Institute of Molecular and Cell Biology, 02-109 Warsaw, Poland and; the § Department of Biochemistry and Functional Proteomics, Institute of Biology II, Faculty of Biology and BIOSS Centre for Biological Signalling Studies, University of Freiburg, 79104 Freiburg, Germany

**Keywords:** membrane, metal, mitochondria, organelle, protein complex, MICOS, Sco1, Cox17, inner membrane architecture

## Abstract

The mitochondrial contact site and cristae organizing system (MICOS) is a recently discovered protein complex that is crucial for establishing and maintaining the proper inner membrane architecture and contacts with the outer membrane of mitochondria. The ways in which the MICOS complex is assembled and its integrity is regulated remain elusive. Here, we report a direct link between Cox17, a protein involved in the assembly of cytochrome *c* oxidase, and the MICOS complex. Cox17 interacts with Mic60, thereby modulating MICOS complex integrity. This interaction does not involve Sco1, a partner of Cox17 in transferring copper ions to cytochrome *c* oxidase. However, the Cox17-MICOS interaction is regulated by copper ions. We propose that Cox17 is a newly identified factor involved in maintaining the architecture of the MICOS complex.

## Teaser

**Background:** MICOS is a recently identified complex that is important for maintaining the architecture of the mitochondrial inner membrane.

**Results:** Cox17 interacts with the MICOS complex, and this interaction is promoted by copper ions.

**Conclusion:** Cox17 regulates MICOS integrity.

**Significance:** Learning the way in which MICOS is regulated provides important insights into processes that are responsible for mitochondrial form and function.

## Introduction

Essential cellular functions of mitochondria are allied with the complex structure of these organelles (1, 2). The two mitochondrial membranes, the inner membrane (IM)^2^ and outer membrane (OM), restrict the boundaries of the two aqueous compartments: the intermembrane space (IMS) and matrix. The IM is divided into the boundary IM, closely juxtaposed to the OM, and invaginations called cristae. Protein transport complexes and other proteins that are involved in communication between the OM and IM are preferentially localized in the inner boundary membrane, whereas cristae are enriched in mature respiratory chain complexes (3–6). Alterations and remodeling of the inner mitochondrial membrane structure are often observed in human pathologies, including neurodegenerative diseases (5, 7). The inner boundary membrane and cristae are joined by structures called crista junctions (8). Distinct groups of proteins are involved in the formation of the IM architecture. The oligomerization of F_1_F_o_-ATPase influences the IM curvature (9). The conserved GTPase from the dynamin family, called Mgm1/OPA1, which catalyzes membrane fusion, is located in crista junctions and regulates both the number of cristae and the release of cristae content (*i.e.* cytochrome *c*) (10–13). The recently identified mitochondrial contact site and cristae organizing system (MICOS) complex is crucial for establishing and maintaining the proper IM architecture. The MICOS complex is composed of six subunits: Mic60, Mic27, Mic26, Mic19, Mic12, and Mic10. Mic60 and Mic10 are the core components of the MICOS complex (14–18). The MICOS complex is localized close to the crista junctions (19). Its dysfunction is caused by the absence of single subunits, particularly the core components Mic60 and Mic10, and leads to the detachment of cristae from the boundary IM (19, 20).

The MICOS complex is also required for the formation of contact sites between the IM and OM to facilitate communication between these two mitochondrial compartments (4, 21, 22). Moreover, the MICOS complex was proposed to form a central core of a large interaction platform that controls the mitochondrial organization system (23). The MICOS complex interacts with the Ugo1 protein, a part of mitochondrial fusion machinery, and the abundant OM channel for small metabolites, porin (15, 16). MICOS components have been found to interact ubiquitously with the sorting and assembly machinery SAM/TOB that is involved in the assembly of β-barrel proteins (24–26). Interactions with the translocase of outer membrane (TOM) complex and mitochondrial IMS import and assembly (MIA) machinery promote the efficient transport of proteins into the IMS of mitochondria (18). Thus, via links with multiple proteins, the MICOS complex functions as an organizer of mitochondrial architecture and integration platform for processes that are centered on mitochondrial membranes.

However, little is known about external factors that regulate the assembly, architecture, and integrity of the MICOS complex. Aim24, a contact site protein, was demonstrated to modulate the MICOS complex, membrane lipid composition, and architecture (27). Here we report that the IMS-located copper chaperone for cytochrome *c* oxidase, Cox17, transiently associates with the MICOS complex and plays a role in modulating its biogenesis and integrity.

## Experimental Procedures

### 

#### 

##### Yeast Strains, Plasmids, and Growth Conditions

The *Saccharomyces cerevisiae* strains that were used in this study were derivatives of YPH499 (*MAT*a, *ade2-101*, *his3*-Δ*200*, *leu2*-Δ*1*, *ura3-52*, *trp1*-Δ*63*, *lys2-801* (28)) and BY4741 (*MAT*a, *his3*Δ *1*, *leu2*Δ *0*, *met15*Δ *0*, *ura3*Δ *0* (Euroscarf, Frankfurt, Germany)). The deletion strains *mic60*Δ (No. 492 in the collection), *mic10*Δ (584), *cox17*Δ (636), and *sco1*Δ (724) were purchased from Euroscarf. The strain that expressed Mic60 with a C-terminal protein A tag (493) was described previously (18). To generate Mic60_ProtA_ *cox17*Δ (878), *cox17*Δ (879) in the YPH499 genetic background, and Mic60_ProtA_ *sco1*Δ (917), the deletion cassette, including the flanking regions, was amplified from genomic DNA of *cox17*Δ or *sco1*Δ and transformed into Mic60_ProtA_ or YPH499 strains using homologous recombination. The plasmid that encoded Cox17 fusion protein (pAG4, 56) with a C-terminal FLAG tag was described previously (29). The yeast media were YPS/YPG (1% (w/v) yeast extract, 2% (w/v) peptone, and 2% (w/v) sucrose/3% (w/v) glycerol, respectively). Strains that were transformed with the pAG4 plasmid or empty vector (pESC-URA) were grown at 28 °C on selective minimal medium that contained 0.5% (w/v) ammonium sulfate and 0.17% (w/v) yeast nitrogen base, supplemented with appropriate nutrients and a carbon source and copper sulfate when indicated. For the drop tests, selective minimal medium that contained 2.5% (w/v) agar was used. The expression of Cox17_FLAG_ was induced for 12 h by the addition of 0.5% galactose to the yeast media. Yeast strains were grown at different temperatures as indicated.

##### Mitochondrial Procedures

The isolation of mitochondria was performed by differential centrifugation according to previously published procedures (30), and mitochondria were resuspended in SM buffer (250 mm sucrose and 10 mm MOPS-KOH, pH 7.2). To analyze the steady-state levels of proteins, mitochondria were solubilized in Laemmli buffer that contained 50 mm DTT (reducing conditions) and denatured by incubation at 65 °C for 15 min. Samples were analyzed by SDS-PAGE followed by Western blot.

##### Affinity Purification of Cox17_FLAG_

Yeast strains that were transformed with the pAG4 plasmid were grown on a selective medium without uracil with 2% (w/v) sucrose or 3% (w/v) glycerol and 0.2% (w/v) sucrose overnight. The expression of Cox17 was induced by incubating with 0.5% (w/v) galactose for 12 h. Yeast cells (300 absorbance units at 600 nm (*A*_600_)) were harvested by centrifugation, resuspended in cold buffer (20 mm Tris-HCl (pH 7.4), 50 mm NaCl, and 50 mm iodoacetamide), and disrupted by a French press (Constant Cell Disruption Systems, CD-019) at a maximum pressure of 31 kpsi. After disruption, 2 mm PMSF and 1% (w/v) digitonin were added, and the cell extracts were solubilized on ice for 20 min. Solubilized material was clarified by centrifugation and incubated with anti-FLAG M2 affinity gel (Sigma-Aldrich) for 1.5 h at 4 °C. The column was washed three times with buffer (20 mm Tris-HCl (pH 7.4), 50 mm NaCl, and 50 mm iodoacetamide). Bound proteins were eluted by incubation in Laemmli buffer with 50 mm DTT. Samples were analyzed by SDS-PAGE followed by Western blot. For mass spectrometric analysis, proteins were affinity-purified from cells that expressed Cox17_FLAG_ or non-tagged endogenous Cox17 (control) eluted with the FLAG peptide (150 μg/ml). Proteins were acetone-precipitated and digested with trypsin. The resulting peptide mixtures were analyzed by nano-high-performance liquid chromatography/electrospray ionization-tandem mass spectrometry on an LTQ-Orbitrap XL instrument (Thermo Fisher Scientific, Bremen, Germany). Raw mass spectrometric data were processed using MaxQuant (version 1.2.7.4) (31, 32) and the *Saccharomyces* Genome Database for protein identification.

The purification of Cox17_FLAG_ was also performed from isolated mitochondria. Yeast strains were grown at 28 °C on YPG medium (1% (w/v) yeast extract, 2% (w/v) Bacto peptone, and 3% (w/v) glycerol). Galactose (0.5% (w/v)) was added to the medium for a 12-h incubation to induce the expression of Cox17_FLAG_. Isolated mitochondria were solubilized in digitonin-containing buffer (1% (w/v) digitonin, 10% (w/v) glycerol, 20 mm Tris-HCl (pH 7.4), 300 mm NaCl, 50 mm iodoacetamide, and 2 mm PMSF) on ice for 20 min. Further affinity purification was performed as described above.

##### Affinity Chromatography of Protein A

For the purification of Mic60 with a C-terminal protein A tag, isolated mitochondria were solubilized with digitonin-containing buffer (1% (w/v) digitonin, 10% (w/v) glycerol, 20 mm Tris-HCl (pH 7.4), 50 mm NaCl, 0.5 mm EDTA, 50 mm iodoacetamide, and 2 mm PMSF) for 20 min on ice. After clarification by centrifugation, extracts were applied on immunoglobulin G-Sepharose (GE Healthcare) and incubated for 1 h at 4 °C. Columns were washed three times (10% (w/v) glycerol, 20 mm Tris-HCl (pH 7.4), 50 mm NaCl, 0.5 mm EDTA, and 2 mm PMSF), and bound proteins were eluted by incubation in Laemmli buffer with 50 mm DTT.

##### Cell Viability Assay

The yeast strains were grown overnight at 24 °C on synthetic minimal medium (0.67% (w/v) yeast nitrogen base and 0.079% (w/v) complete supplement mixture of amino acids) that contained a respiratory carbon source (3% (w/v) glycerol) and supplemented with different concentrations of copper sulfate. One *A*_600_ unit of yeast cells was harvested by centrifugation, and the pellet was washed with PBS (137 mm NaCl, 12 mm phosphate, and 2.7 mm KCl, pH 7.4). To assess cell viability, propidium iodide (3 μg/ml) was added to cells that were suspended in PBS and incubated for 15 min at room temperature while protected from light. Samples were kept on ice and analyzed within 1 h by flow cytometry (FACSCalibur). For each sample, 30,000 cells were measured.

##### Miscellaneous

The experiments were performed usually in at least three (a minimum of two) biological repetitions and were additionally controlled by technical replicates. Cellular total protein extracts were prepared from yeast cells using alkaline lysis (33). Protein samples were analyzed on 15% acrylamide gels. Western blot was performed according to a semi-dry transfer procedure using a polyvinylidene difluoride membrane (Millipore). The primary antibodies that were used in this study were raised in rabbits, with the exception of the anti-FLAG M2 (Stratagene) antibody, which was raised in mice. In some figures, non-relevant gel parts were excised digitally. The nomenclature for the proteins is according to the *Saccharomyces* Genome Database. The signals from enhanced chemiluminescence were detected with x-ray film (Foton-Bis). Protein concentrations were determined according to the Bradford method with bovine serum albumin as the standard.

## Results

### 

#### 

##### MICOS Complex Components Interact with Cox17

**FIGURE 1.**
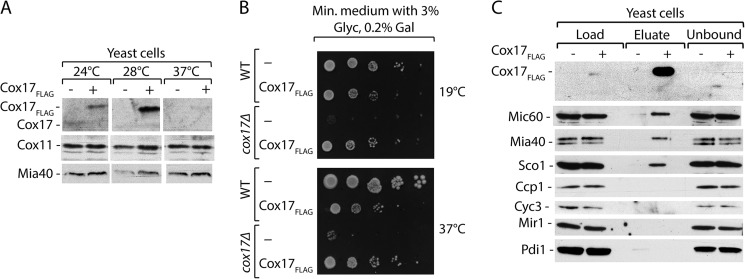
**Characterization of Cox17_FLAG_ as a model protein to study interactions.** *A*, cellular protein extracts of cells expressing Cox17_FLAG_ at different temperatures. Proteins were separated by SDS-PAGE and analyzed by Western blotting. *B*, wild-type and *cox17*Δ strains expressing Cox17_FLAG_ were subjected to consecutive 10-fold dilutions, spotted on selective minimal medium (*Min. medium*) containing 3% glycerol and 0.2% galactose, and grown at the indicated temperatures. *C*, affinity purification of Cox17_FLAG_ upon cell disruption and solubilization with digitonin. Load, 3%; eluate, 100%.

Cox17 is a small IMS protein that contains the C*X*_9_*C* motif and requires the MIA pathway for its import and assembly (34, 35). Its function is essential for respiration because Cox17 acts as a metal chaperone by delivering copper ions to Sco1 and Cox11 and ultimately to cytochrome *c* oxidase (36–38). To identify the interaction partners of Cox17, we used the fusion protein of Cox17 with a FLAG tag at the C terminus under the inducible *GAL10* promoter (29, 39). We found that 28 °C was the optimal temperature for Cox17_FLAG_ production (Fig. 1*A*). The *cox17*Δ cells do not grow on the respiratory medium (36, 37). To assess the functionality of Cox17_FLAG_, we tested the respiratory growth of cells that produce Cox17_FLAG_. Cox17_FLAG_ led to a slight growth retardation of wild-type cells at 37 °C (Fig. 1*B*). However, it restored the growth of the *cox17*Δ strain on the respiratory medium at both temperatures, 19 °C and 37 °C (Fig. 1*B*). Thus, Cox17_FLAG_ complemented the function of its native equivalent and is an appropriate model to study protein interactions.

To identify Cox17-interacting partners, we affinity-purified the protein from extracts of cells that express Cox17_FLAG_ or control cells that express non-tagged endogenous Cox17 and analyzed the samples by mass spectrometry. Surprisingly, among the proteins highly enriched in the Cox17_FLAG_ eluate, we found Mic60, which is a core component of the MICOS complex. We analyzed the elution fraction of Cox17_FLAG_ with specific antibodies (Fig. 1*C*). We found Mic60 in the elution fraction. Mia40, a core component of MIA machinery, is responsible for the transport and oxidative folding of Cox17, and thus it interacts with Cox17 (34). The transient *in vivo* interaction between Cox17_FLAG_ and Mia40 was observed previously (29) and was confirmed in the present experiment (Fig. 1*C*), thus demonstrating the sensitivity of the assay. Interestingly, we were able to co-purify Sco1, a protein that transiently cooperates with Cox17 in the delivery of copper ions to cytochrome *c* oxidase (38, 40, 41). Several mitochondrial proteins (Ccp1, Cyc3, and Mir1) and a non-mitochondrial protein (Pdi1 from the endoplasmic reticulum) were not detected in the elution fraction, confirming the specificity of the assay. Thus, Mic60 is a newly identified interaction partner of Cox17.

**FIGURE 2.**
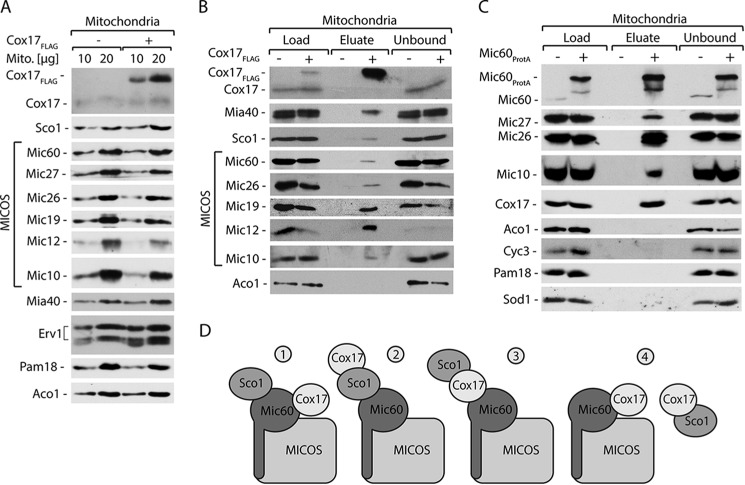
**Cox17 interacts with the MICOS complex.** *A*, steady-state protein levels of mitochondria (*Mito.*) isolated from cells expressing Cox17_FLAG_. *B*, affinity purification of Cox17_FLAG_ upon solubilization of mitochondria with digitonin. *C*, affinity purification of Mic60_ProtA_ from the isolated mitochondria upon solubilization with digitonin. *B* and *C*, load, 3%; eluate, 100%. *A–C*, proteins were separated by SDS-PAGE and analyzed by Western blotting. *D*, schematic representation of hypothetical complexes formed by Cox17 with its interacting partners.

To determine whether Cox17 interacts with other MICOS components, we isolated mitochondria from cells that produced Cox17_FLAG_. The steady-state levels of mitochondrial proteins, including Sco1, and MICOS components were unaffected upon Cox17_FLAG_ expression (Fig. 2*A*). We performed affinity purification using these isolated mitochondria (Fig. 2*B*). The result of this experiment confirmed that Cox17_FLAG_ interacts with Mia40 and Sco1. In addition to Mic60, we co-purified other MICOS complex components, including Mic26, Mic19, Mic12, and Mic10 (Fig. 2*B*). Aco1 served as a negative control (Fig. 2*B*). This experiment showed that Cox17 interacts not only with Mic60 but also with the entire MICOS complex.

To exclude the possibility that the interaction between Cox17 and MICOS was caused by the overexpression of Cox17_FLAG_ fusion protein, we performed affinity purification using a reverse experimental setup that utilized Mic60_ProtA_ as a bait to look for native Cox17 as a prey. Cells with Mic60_ProtA_ (18) were used for mitochondrial isolation and subsequent affinity purification (Fig. 2*C*). These experiments showed that Mic60 interacted with the native form of Cox17, in addition to other MICOS components (Fig. 2*C*). These results confirmed our previous observation concerning the specificity of the Cox17 interaction with the MICOS complex (Figs. 1*C* and 2*B*). Thus, native Cox17 also interacts with the MICOS complex in a transient manner.

Based on these results, we proposed four possible models for Cox17 contacts with the detected partners (Fig. 2*D*). According to the first model, Cox17 interacts with Sco1 indirectly via Mic60 or the MICOS complex. The second model proposes that Cox17 interacts with the MICOS complex via Sco1 protein. According to the third model, Cox17 mediates the interaction of the MICOS and Sco1. The fourth model proposes that Cox17 forms two separate complexes with Sco1 or the MICOS complex. Our next attempts were directed toward identifying the precise mode of Cox17 interactions (Fig. 2*D*).

##### Interactions of Cox17 with Sco1 and MICOS Are Independent

**FIGURE 3.**
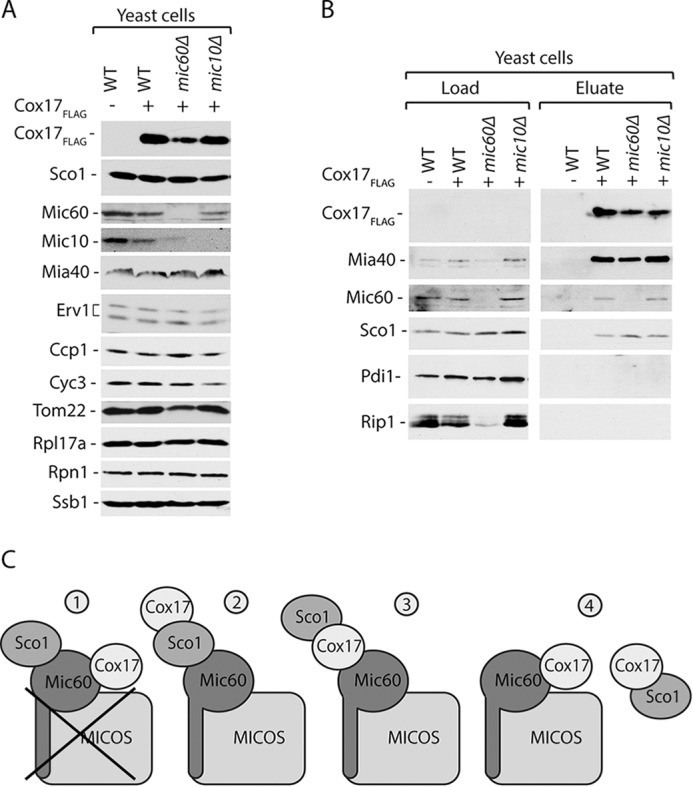
**Interaction of Cox17 with Sco1 does not depend on MICOS.** *A*, protein levels in wild-type, *mic60*Δ, and *mic10*Δ cells expressing Cox17_FLAG_. *B*, affinity purification of Cox17_FLAG_ upon cell disruption and solubilization with digitonin. Load, 3%; eluate, 100%. Proteins were separated by SDS-PAGE and analyzed by Western blotting. *C*, the interaction between Cox17 and Sco1 is not via MICOS.

We analyzed the Cox17_FLAG_ interaction with Sco1 in the deletion strains of Mic60 and Mic10. In the absence of Mic60 or Mic10, the MICOS complex is disrupted and non-functional (14–16, 18, 42). We produced Cox17_FLAG_ in *mic60*Δ and *mic10*Δ cells. Immunoblotting confirmed the deletion of *mic60* and *mic10* and reduction of Mic10 levels in *mic60*Δ cells (Fig. 3*A*) as reported earlier (15, 18). The levels of Sco1, Mic60, Mic10, and MIA pathway components (*i.e.* Mia40 and Erv1) and other unrelated mitochondrial and cellular proteins were unaffected upon overexpression of Cox17_FLAG_ (Fig. 3*A*). Affinity purification showed that Mia40, which was used as a positive control, was efficiently co-isolated with Cox17_FLAG_ from the protein extracts of wild-type cells and *mic60*Δ and *mic10*Δ cells (Fig. 3*B*). Thus, the interaction between Cox17 and Mia40 did not depend on the MICOS complex. Mic60 was also co-isolated with Cox17_FLAG_, even in the absence of Mic10 (Fig. 3*B*), when the MICOS complex is destabilized (18). This result indicates that destabilization of the MICOS complex does not affect the Cox17-Mic60 interaction and suggests that Cox17 binds to Mic60 and not other MICOS components. Moreover, despite the deletion of Mic60 and Mic10, Sco1 co-purified with Cox17_FLAG_ (Fig. 3*B*), thus excluding the possibility that Cox17 forms a complex with Sco1 via Mic60 or via the MICOS complex (Fig. 3*C*).

**FIGURE 4.**
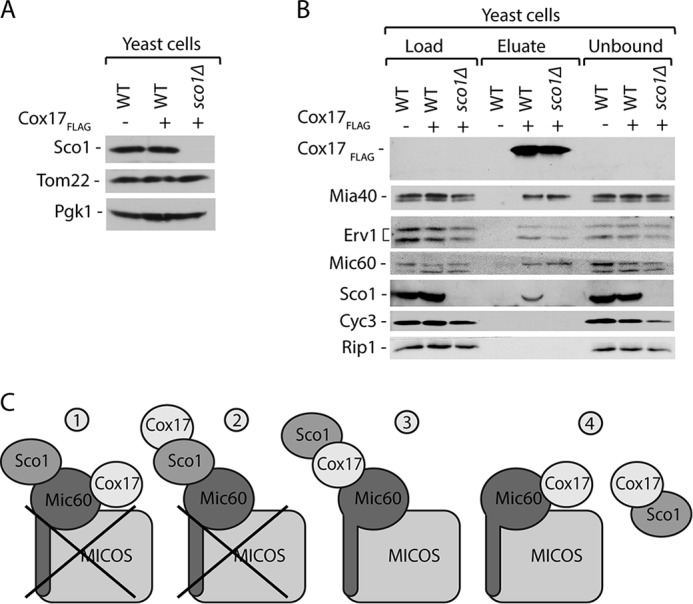
**Cox17 interaction with Mic60 does not depend on Sco1.** *A*, protein levels in wild-type and *sco1*Δ strains expressing Cox17_FLAG_. *B*, affinity purification of Cox17_FLAG_ upon cell disruption and solubilization with digitonin. Load, 3%; eluate, 100%. Proteins were separated by SDS-PAGE and analyzed by Western blotting. *C*, the interaction between Cox17 and Mic60 is not via Sco1.

We also examined the second model, which proposes that the Cox17 interaction with the MICOS complex occurs via Sco1 (Figs. 2*D* and 3*C*). We evaluated the possibility that Cox17 interacts with Mic60 via Sco1 using the affinity purification of Cox17_FLAG_ from cells that lacked Sco1 (*i.e. sco1*Δ cells; Fig. 4*A*). The lack of Sco1 did not influence the Cox17 interaction with Mia40 and Erv1 (Fig. 4*B*) (29). Furthermore, the deletion of Sco1 did not affect the interaction between Mic60 and Cox17 (Fig. 4*B*). These findings suggest that Cox17 does not form a complex with Mic60 via Sco1, enabling us to exclude the second model (Fig. 4*C*).

**FIGURE 5.**
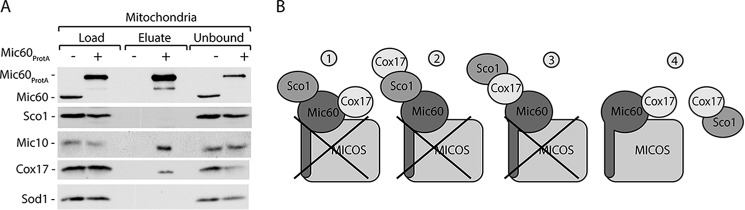
**Sco1 does not interact with Mic60.** *A*, affinity purification of mitochondria isolated from cells expressing Mic60_ProtA_ upon solubilization with digitonin. Load, 3%; eluate, 100%. Proteins were separated by SDS-PAGE and analyzed by Western blotting. *B*, Cox17 forms two assemblies, either with Sco1 or with MICOS.

We addressed the possibility of Cox17 being in the central position between Sco1 and Mic60 using affinity purification of Mic60_ProtA_ (Fig. 5*A*). Sco1 did not co-purify with Mic60_ProtA_, in contrast to Mic10 and Cox17 (Fig. 5*A*). Thus, Sco1 does not form a complex with Mic60 or MICOS (Fig. 5*B*). Altogether, our results indicate that Cox17 forms two independent complexes with MICOS and Sco1 (Fig. 5*B*).

**FIGURE 6.**
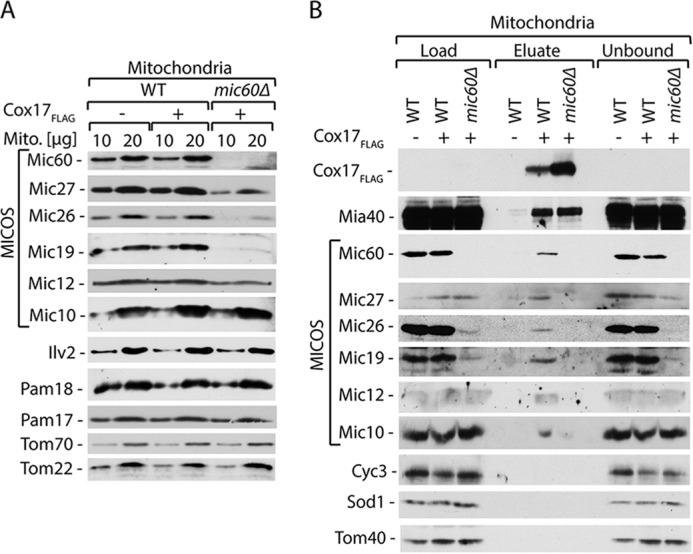
**Cox17 interacts with MICOS via Mic60.** *A*, steady-state protein levels of mitochondria (*Mito.*) isolated from wild-type or *mic60*Δ strains expressing Cox17_FLAG_. *B*, affinity purification of Cox17_FLAG_ upon solubilization of mitochondria with digitonin. Load, 3%; eluate, 100%. Proteins were separated by SDS-PAGE and analyzed by Western blotting.

Cox17 interaction with Mic60 was maintained in the *mic10*Δ cells despite MICOS destabilization (Fig. 3*A*). To analyze the Cox17 interaction with MICOS in yeast strains that lack Mic60, we isolated mitochondria from *mic60*Δ cells that expressed Cox17_FLAG_. The steady-state levels of mitochondrial proteins were determined, including MICOS complex components (Fig. 6*A*). As expected, the expression of Cox17_FLAG_ did not affect the levels of MICOS complex components in wild-type mitochondria. The deletion of Mic60 resulted in almost complete loss of Mic26 and Mic19 and a decrease in Mic27 and Mic12, whereas the levels of Mic10 remained unchanged as compared with wild-type cells (Fig. 6*A*). This is consistent with previously published results (18). We performed affinity purification via Cox17_FLAG_ (Fig. 6*B*). In the case of wild-type mitochondria, we found MICOS complex components in the eluate together with Cox17. We did not find Mic26 or Mic19 in the *mic60*Δ eluate because the steady-state levels of these proteins in mitochondria were severely affected. Importantly, we noticed loss of the interaction between Cox17 and Mic10, Mic12, and Mic27 (Fig. 6*B*), for which the steady-state protein levels were unchanged or weakly affected (Fig. 6*A*). The control proteins, including Cyc3, Sod1, and Tom40, were not found in the eluate (Fig. 6*B*). These results demonstrate that Mic60 is required for the interaction between Cox17 and the MICOS complex and that Mic60 mediates this interaction.

##### Cox17 Promotes MICOS Complex Integrity

**FIGURE 7.**
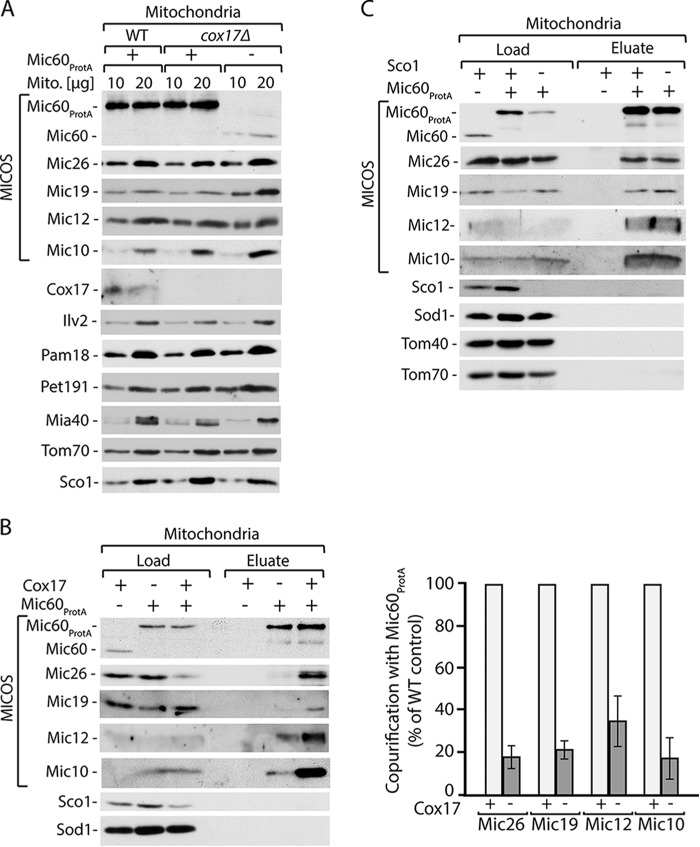
**Cox17 deletion affects the MICOS integrity.** *A*, steady-state protein levels of mitochondria (*Mito.*) isolated from Mic60_ProtA_, Mic60_ProtA_ *cox17*Δ, and *cox17*Δ strains. *B*, affinity purification of Mic60_ProtA_ from the *cox17*Δ and wild-type mitochondria upon solubilization with digitonin. MICOS complex components co-purified with Mic60 were quantified and normalized to the efficiency of Mic60_ProtA_ recovery. Recovery of MICOS components was set to 100%. Data are represented as mean ± S.E. (*n* = 3); *n* used to calculate S.E. refers to biological repetitions (different mitochondria preparations). *C*, affinity purification of Mic60_ProtA_ from the *sco1*Δ and wild-type mitochondria upon solubilization with digitonin. *B and C*, load, 3%; eluate, 100%. *A–C*, cells grown under fermentative conditions on sucrose as a carbon source. Proteins were separated by SDS-PAGE and analyzed by Western blotting.

To assess the role of Cox17 in the MICOS complex, we examined MICOS complex integrity. We generated a yeast strain that expressed Mic60 C-terminally tagged with protein A and harbored the *COX17* gene deletion. Because Cox17 is required for the assembly of cytochrome *c* oxidase, cells that lack Cox17 do not grow on the respiratory medium (36, 37) (Fig. 1*B*). Thus, we grew our strains in the medium supplemented with a fermentative carbon source, sucrose. Under these conditions, the levels of MICOS complex subunits were unaffected in *cox17*Δ cells as compared with wild-type cells, which both expressed Mic60_ProtA_ (Fig. 7*A*). We performed affinity purification via Mic60_ProtA_. In the mitochondria that were isolated from yeast cells lacking Cox17, the interaction of Mic60_ProtA_ with Mic26, Mic19, Mic12, and Mic10 was severely decreased as compared with the wild-type strain (Fig. 7*B*). This reflects dissociation of the MICOS complex in the *cox17*Δ strain. The possibility existed that the destabilization of the MICOS complex is caused in an unspecific manner by defects in the assembly of cytochrome *c* oxidase and respiratory deficiency. Thus, we performed affinity purification via Mic60_ProtA_ from the mitochondria of yeast cells that lacked Sco1 protein (Fig. 7*C*). The interaction of Mic60_ProtA_ with Mic26, Mic19, Mic12, and Mic10 was not affected by the lack of Sco1. Our findings support the conclusion that Cox17 plays an important role in modulating MICOS complex integrity.

**FIGURE 8.**
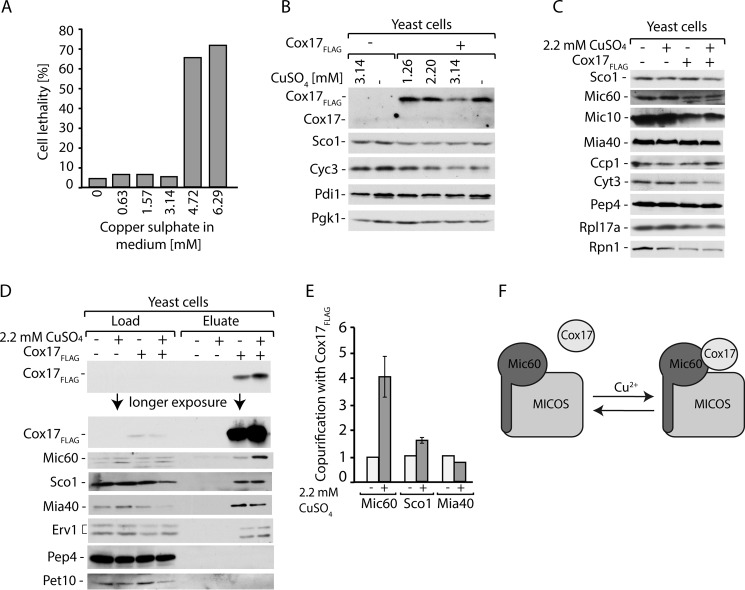
**Copper ions increase the interaction of Cox17 with Mic60.** *A*, the cell lethality after the addition of different concentrations of copper sulfate to the medium assessed by propidium iodide staining. *B*, Cox17_FLAG_ levels in cells grown on the medium supplemented with different concentration of copper sulfate. *C*, protein levels in wild-type cells expressing Cox17_FLAG_. Cells were grown on medium with or without 2.2 mm copper sulfate. *D*, affinity purification of Cox17_FLAG_ upon cell disruption and solubilization with digitonin. Load, 3%; eluate, 100%. Proteins were separated by SDS-PAGE and analyzed by Western blotting. *E*, quantitative analysis of Cox17 interactions with Mic60, Sco1, and Mia40 upon growth of cells in the presence of 2.2 mm copper sulfate after normalization to the expression levels of the proteins in the load fraction and the efficiency of Cox17_FLAG_ recovery. Data are represented in -fold change as compared with untreated cells. Mean ± S.E. (*n* = 3); *n* used to calculate S.E. refers to independent biological replicates. *F*, schematic representation of Cox17 interaction with MICOS supported by copper ions.

Cox17 is a copper chaperone that is involved in the transfer of copper ions (36–38, 43). We sought to analyze the effect of copper ions on the interaction between Cox17 and Mic60. We first tested the concentration of copper ions in the growth medium to exclude lethal or sublethal doses (Fig. 8*A*). Copper sulfate concentrations greater than 4 mm in the medium were lethal for yeast cells under the tested conditions. Furthermore, we analyzed whether the addition of copper ions to the medium influences the expression of Cox17_FLAG_ (Fig. 8*B*). The addition of copper sulfate concentrations greater than 3 mm led to a decrease in Cox17_FLAG_ expression, whereas the 2.2 mm concentration resulted in the same expression levels of Cox17_FLAG_ as in the untreated cells (Fig. 8*B*). We decided to use the 2.2 mm concentration of copper sulfate in the subsequent experiments. We grew wild-type and Cox17_FLAG_-producing cells in the presence or absence of copper sulfate and determined the levels of the mitochondrial proteins Sco1, Mic60, and Mic10 and non-mitochondrial proteins Pep4, Rpl17a, and Rpn1 (Fig. 8*C*). The levels of all tested proteins were unchanged upon growth in the presence of copper sulfate and the expression of Cox17_FLAG_ (Fig. 8*C*). To investigate the influence of copper ions on the interaction between Cox17 and Mic60, we affinity-purified Cox17_FLAG_ from yeast cells that were grown in the presence or absence of copper sulfate (Fig. 8*D*). Mic60 was co-purified with Cox17 more efficiently when the cells were grown in the presence of copper ion addition. We quantified the levels of Mic60, Sco1, and Mia40 that were co-isolated with Cox17_FLAG_ (Fig. 8*E*). The interaction with Mia40 was unaffected by the presence of copper ions. The interaction between Cox17 and Sco1 was slightly more efficient. In agreement with Fig. 8*D*, quantification confirmed that the interaction between Cox17 and Mic60 markedly increased upon the addition of copper ions to the medium (Fig. 8*E*). This observation supports the hypothesis that copper ions promote the Cox17 interaction with MICOS via Mic60 (Fig. 8*F*).

## Discussion

The present study identifies Cox17 as a novel factor involved in regulating the MICOS complex. Cox17 forms two independent assemblies, one with Sco1 for the biogenesis of cytochrome *c* oxidase (38), and another one with the MICOS complex. The latter association promotes MICOS complex integrity. This finding places Cox17 together with Aim24 (27) in a novel group of proteins that regulate the biogenesis and/or stability of MICOS. To perform its function, Aim24 interacts with the IM-embedded Mic10 (27), whereas Cox17 contacts the IMS-exposed Mic60 component of MICOS. Hypothetically, through direct binding, Cox17 may control the assembly of other subunits of the MICOS complex with Mic60. This possibility is supported by the fact that Mic60 is a core subunit of the MICOS complex, and its absence leads to reduction of the entire complex (18).

MICOS complex regulation by Cox17 is promoted by copper ions. Two plausible scenarios can be considered to envision the new copper ion-dependent function of Cox17 in regulating MICOS. First, Cox17 may play a role in the assembly or stabilization of the MICOS complex. This Cox17 function would be promoted and would depend on the ability to bind copper ions. Alternatively, Cox17 may directly facilitate the delivery of copper ions to the MICOS complex. The presence of copper ions may regulate the levels of mature MICOS. In summary, Cox17 is not only involved in the biogenesis of respiratory complexes but also is the auxiliary factor that controls the MICOS complex that is crucial for mitochondrial membrane organization.

## Footnotes

2 The abbreviations used are:

used: IM: inner membrane
IMS: intermembrane space
OM: outer membrane
MIA: mitochondrial IMS import and assembly
MICOS: mitochondrial contact site and cristae organizing system
ProtA: protein A.
